# Threonine Facilitates Cd Excretion by Increasing the Abundance of Gut *Escherichia coli* in Cd-Exposed Mice

**DOI:** 10.3390/molecules28010177

**Published:** 2022-12-25

**Authors:** Yongbin Li, Zhijia Fang, Xuewei Zhou, Jian Gao, Jingwen Wang, Linru Huang, Yinyan Chen, Lijun Sun, Qi Deng, Ravi Gooneratne

**Affiliations:** 1 College of Food Science and Technology, Guangdong Provincial Key Laboratory of Aquatic Product Processing and Safety, Guangdong Provincial Engineering Technology, Research Center of Marine Food, Key Laboratory of Advanced Processing of Aquatic Products of Guangdong Higher Education Institution, Guangdong Ocean University, Zhanjiang 524088, China; 2 College of Continuing Education, Guangdong Ocean University, Zhanjiang 524088, China; 3 Department of Wine, Food and Molecular Biosciences, Lincoln University, Lincoln, Canterbury 7647, New Zealand

**Keywords:** cadmium, amino acids, threonine, *Escherichia coli*, gut microbiota

## Abstract

Cadmium (Cd) can easily enter the body through the food chain and threaten health since Cd pollution is prevalent in the environment. Gut microbiota is necessary for the reduction of metal ions. To reduce Cd-induced harmful impacts and Cd accumulation in the body, we investigated the effect of amino acids on gut microbiota and Cd excretion in (fecal Cd) Cd-exposed mice. The screening of 20 amino acids showed that threonine (Thr) effectively increased fecal Cd, and reduced Cd-induced intestinal structural damage. The abundance of *Escherichia-Shigella* genus and KF843036_g significantly increased after the oral administration of Thr. As the type species of the *Escherichia-Shigella* genus, *Escherichia coli* exhibited high similarity to KF843036_g species and significantly decreased Cd-induced gut damage. Cd contents in the liver, kidney, and gut of Cd-exposed mice were also significantly (*p* < 0.05) decreased after *E. coli* treatment, while the contents in the feces were increased. The results demonstrated the potential roles that gut *E. coli* might play in Thr-mediated Cd excretion in Cd-exposed mice. The findings may provide important data for better understanding the molecular biological mechanism of Thr in reducing Cd accumulation in the body.

## 1. Introduction

Cadmium (Cd) pollution is considerably widespread in the food chain due to human activities, including the use of phosphate fertilizers and industrial activities [1]. Cd can easily accumulate in the human body as a result of Cd exposure, causing various diseases [2,3,4,5]. The elimination of Cd in vivo is difficult to achieve in a short period of time [6]. Continuous exposure to Cd may adversely impact fertility, musculoskeletal diseases, and even cancer development [2,3,4,5]. Dietary intake is the main route of human Cd exposure. Research indicates that vegetables, meat, egg products, and fruits in China contain Cd [7,8,9]. A study of Cd exposure among children, conducted by the Food and Drug Administration (FDA) in 2014–2016, reported that the highest mean concentration of Cd (375 μg/kg) was in sunflower seeds, making the consumption of sunflower seeds a great concern [10]. Most consumed Cd is initially absorbed in the gut before transportation to other organs [11,12]. He et al. [13] studied drinking-water Cd exposure in mice and observed intestinal villi shedding, decreased expression of intestinal tight junction genes, and increased levels of the cytokine TNF-α. Cd exposure in mice also significantly decreases gut microbial richness and inhibits the gene pathways related to the metabolism of amino acids (AAs) [13,14,15].

The gut microbiota (GM) provides essential modulation of immunity, metabolism, adiposity, homeostasis, and energy balance [16,17,18]. As part of the intestinal barrier, GM is closely related to gut homeostasis, and gut dysbacteriosis can potentially lead to compromised gut defense [19,20]. GM secretes bioactive compounds that contribute to various functions, such as anti-virulence and metal chelation [21,22,23]. GM can also restore the intestinal barrier and reduce the bioaccessibility of heavy metals by binding to or transforming metals [24,25]. Due to its binding affinity, exposure to Cd can disturb the homeostasis of the GM [26]. The abundance of the *Clostridium*, *Lactobacillus*, *Pseudomonas*, and *Enterobacterium* genus decreased in the guts of Cd-exposed mice [26]. GM also plays a significant role in inhibiting the absorption of Cd in mice [22,27]. Previous studies have described the use of probiotics such as *Lactobacillus plantarum* HD 48 and CCFM8610 to reduce the level of Cd in the gut [22,25,28].

Dietary supplements have a protective effect against Cd stress [29]. Dietary components such as CaCl_2_ and proanthocyanidins can decrease the bioaccessibility of Cd in rice [30,31]. Likewise, our previous study indicated that AAs have strong protective effects against Cd toxicity in yeast [32]. AAs are prevalent nutrients that can regulate the metabolic function of mice against heavy metal stress [33,34]. In addition, AAs can increase Cd resistance and reduce Cd accumulation in plants [35]. Glycine is reported to inhibit Cd-induced effects by inhibiting the placental transport of Cd [36]. The AA taurine increases the fecal Cd level in mice [37]. Moreover, Cd exposure causes changes in the metabolic function of the gut microbiome and inhibits gene pathways associated with AA metabolism [13]. AAs are capable of modulating the GM in the colon [38]. Additionally, chelating agents can effectively remove Cd from the body [39]. The high excretion of Cd facilitated by the GM may be associated with the Cd chelating ability of the GM [25]. Since GM can impede the adsorption of Cd in mice [22], AAs may play a role in regulating and stabilizing the cumulative Cd toxicity in mice.

However, the role of AAs in the interaction between GM and Cd reduction is unclear, and the mechanism by which the Cd residues in the body is not fully understood. The main goals of the present study were to: (1) evaluate the protective effects of 20 AAs on Cd-exposed mice from the perspective of the GM and identify the key microorganisms that contribute to these effects; (2) verify the contributions of key microorganisms in vivo and identify the mechanism by which the key microorganisms facilitate Cd excretion. These results will contribute to an improved understanding of the roles of AAs in protecting mice under Cd exposure via GM and may provide an effective means for reducing Cd accumulation in the body.

## 2. Results

### 2.1. Effects of AAs on Cd Levels and GM in Cd-Exposed Mice

**Figure 1:**
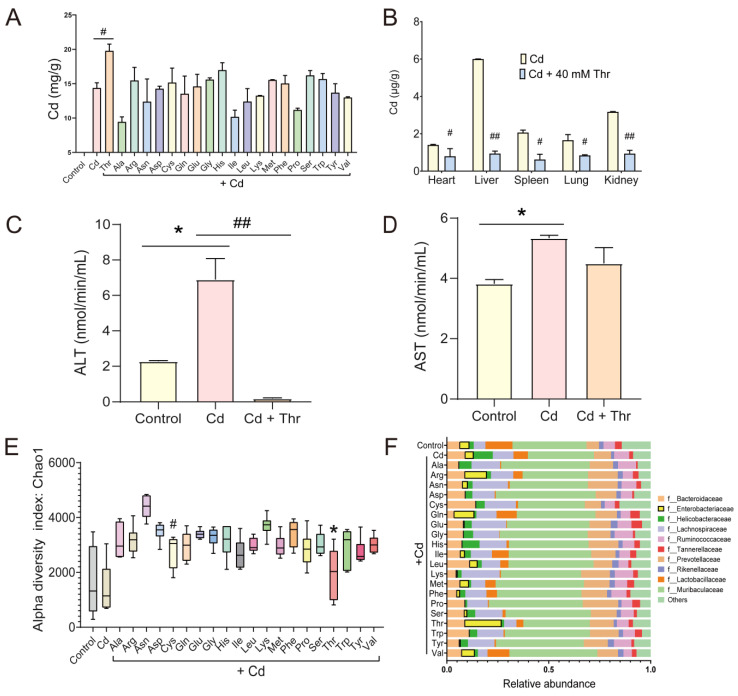
Effects of different amino acids (AAs) on Cd level and gut microbiota (GM) in mice. (**A**) Cd contents in feces. (**B**) Cd contents in tissues. (**C**) Serum alanine aminotransferase (ALT) level. (**D**) Serum aspartate aminotransferase (AST) level. (**E**) The microbiome community richness index Chao of the GM. (**F**) The relative abundance of the top 10 abundances bacterial phyla at the family level. ^#^ *p* < 0.05, ^##^ *p* < 0.001 indicate results that differ significantly from the Cd-treated group. * *p* < 0.05 indicate results that differ significantly from the control group.

To gain insight into the effects of AAs on the Cd-exposed mice, 20 AAs were orally administrated to assess the potential Cd excretion. Cd excretion in Cd-exposed mice was evaluated via fecal Cd content. The average levels of Cd in the feces are presented in Figure 1A. In comparison to the control group, the oral administration of Thr significantly increased the fecal Cd levels. By contrast, there was no significant increase in fecal Cd levels with oral administration of the other AAs (Figure 1A). The levels of Cd were significantly decreased in the heart, liver, spleen, lung, and kidney in the Thr-treated group (Figure 1B). In addition, Cd-induced serum alanine aminotransferase (ALT) and aspartate aminotransferase (AST) levels descended with the treatment of Thr (Figure 1C,D). These results indicate that oral Thr affects Cd accumulation and may facilitate Cd excretion in mice.

Homeostasis of the GM with oral administration of AAs was analyzed by high-throughput 16S rDNA sequencing. The Chao index, an index of microbiome community richness, evinces that Alpha diversity declined slightly with exposure to Cd (Figure 1E). The Chao index of the Thr-treated mice was restored to a level similar to the control group (with similar upper and lower quartiles) (Figure 1E). The GM composition profiles at the family level indicated that Cd exposure significantly decreased the proportions of *Tannerellaceae*, *Rikenellaceae*, *Lachnospiraceae*, *Prevotellaceae*, *Lactobacillaceae*, and *Enterobacteriaceae* in the GM in comparison to the control group (Figure 1F). However, the oral administration of Thr significantly restored the proportion of *Enterobacteriaceae* (Figure 1F). The high level of fecal Cd could be ascribed to the protective effect of AAs on these key GMs that are inhibited by Cd. The results imply that Thr may promote the excretion of Cd by restoring the GM.

**Figure 2:**
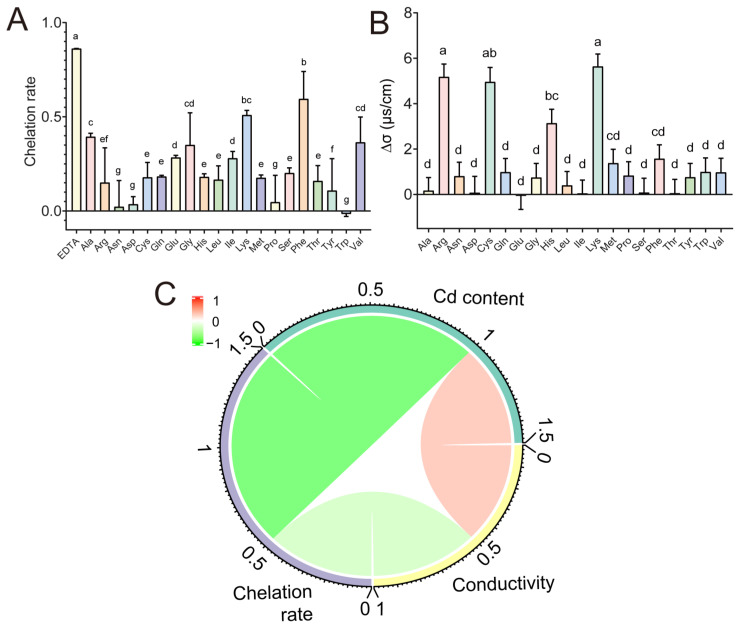
Determination of the Cd chelating ability of 20 AAs. (**A**) CAS assay for Cd chelating features of 20 AAs. (**B**) Changes in conductivity are caused by metal chelating ability. (**C**) Spearman’s correlations among content, conductivity, and chelation rate. Different superscript letters indicate significantly different at *p* < 0.05 among AAs-treated groups.

The Cd chelation abilities of 20 AAs were evaluated to determine whether the high Cd excretion was caused by the ability of AAs to chelate Cd in the gut. The binding characteristics of Cd and the AAs were evaluated according to the conductivity difference (∆σ). Arginine (Arg), cysteine (Cys), histidine (His), and lysine (Lys) exhibited stronger Cd binding abilities but induced less Cd excretion in the feces when compared to Thr (Figure 1A and Figure 2). Another approach to determining the Cd chelating abilities of AAs is through the modified Chrome azurol S (CAS) liquid assay. As shown in Figure 2A, the chelation rate of the EDTA was significantly higher than others in the CAS solution, which indicates that EDTA had a strong Cd chelating ability. phenylalanine (Phe) and Lys exhibited high Cd binding abilities in the CAS assay but produced less Cd excretion when compared to threonine (Thr) (Figure 2A,B). These findings suggested that the Cd chelating ability of AAs may not be related to the Cd excretion in feces. Hence, to test the correlation between Cd chelation and Cd excretion, Spearman’s correlation analysis was performed. The results manifest that Cd excretion was not significantly correlated with the conductivity difference nor with the results of the CAS assay (Figure 2C), indicating that Cd excretion was not induced by Cd chelation. Thus, the underlying mechanism remained unclear.

### 2.2. Thr Restored the Proportion of Escherichia-Shigella in GM of Cd-Exposed Mice

**Figure 3:**
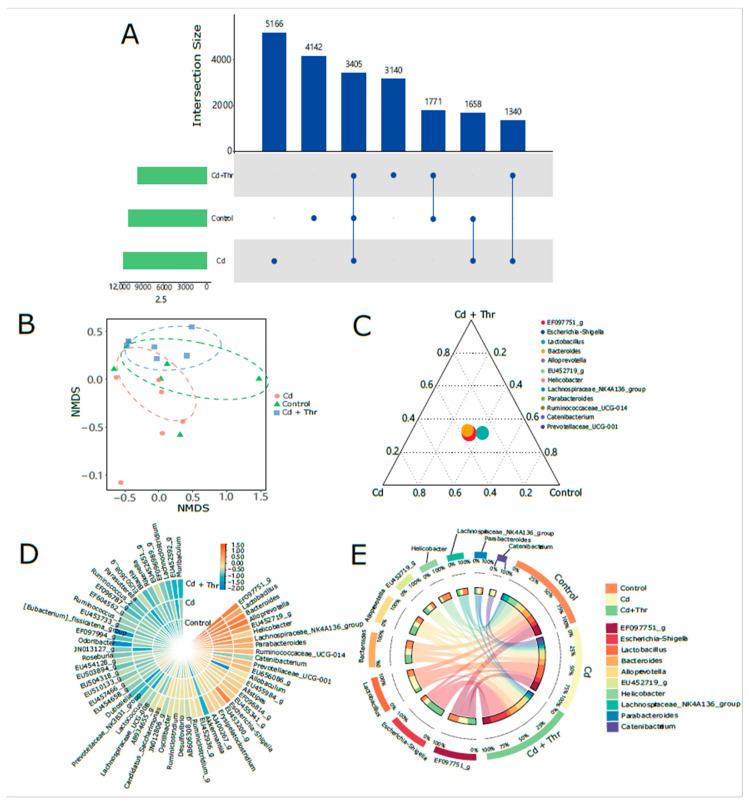
Effects of Thr on GM in mice. (**A**) The result of 97% similarity of operational taxonomic units (OTUs) was described in the UpSet diagram of the control, Thr-treated, and Cd-treated groups. The dotted circles beneath the bar chart indicate the group that contains the same OTU. (**B**) The differences in microbiota structure were described in non-metric multidimensional scaling analysis (NMDS) maps. The distance between the two communities was specified using the weighing of UniFrac. (**C**) The gut microbial component at the genus level was analyzed with a Ternary Plot diagram. (**D**) The microbial abundance heatmap at the genus level. (**E**) Circos graph represented the composition of GM at the genus level. The abundance of the top 10 bacteria was chosen to demonstrate the structure of GM.

Next, we analyzed the characteristics of the GM of Cd-exposed mice after Thr treatment (Figure 3). The number of shared operational taxonomic units (OTUs) between the Thr-treated and control groups was higher than that between the control and Cd-treated groups (Figure 3A). The non-metric multidimensional scaling analysis (NMDS) maps showed that the overlapping regions between the Thr-treated and control groups were bigger than those between the Cd-treated and control groups (Figure 3B). The UpSet diagram and NMDS analysis results implied that Thr plays a protective role on the GM against Cd exposure. The *Escherichia-Shigella* genus was significantly restored in Cd-exposed mice after Thr treatment, as shown in the microbiome heatmaps (Figure 3C). The abundance profiles, ternary plot diagrams, and circos graph demonstrate that the proportion of *Escherichia-Shigella* significantly increased after oral administration of Thr (Figure 3C,D,E). These results indicate that Cd reduction may be associated with gut *Escherichia-Shigella* in Thr-treated mice.

**Figure 4:**
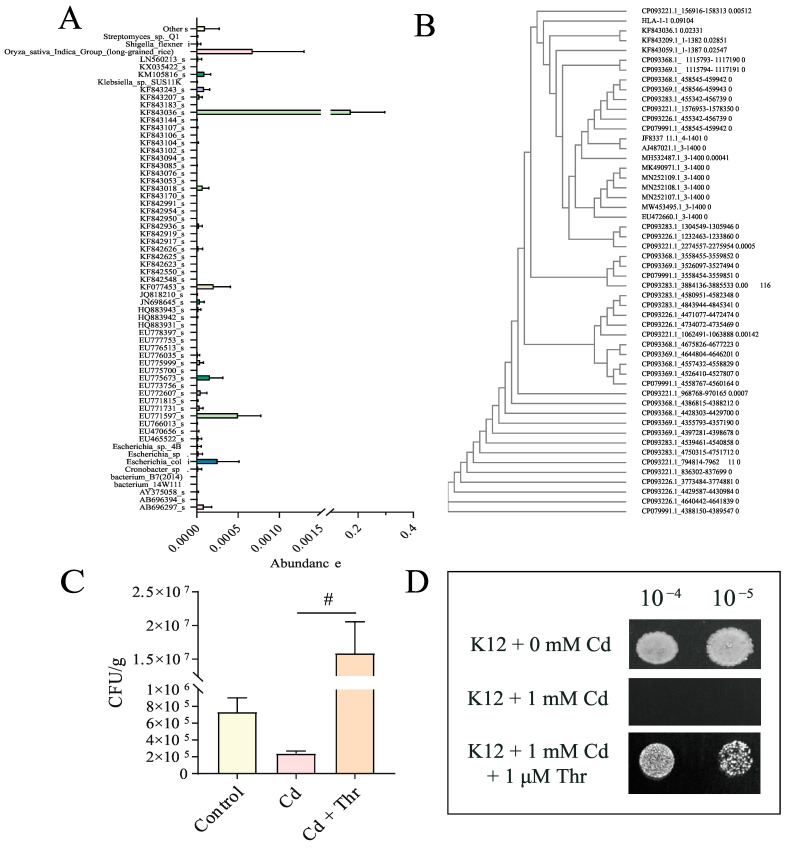
Identification of *E. coli*. (**A**) Average enumeration of *E. coli* in mice feces of 5 days. (**B**) Species component and the correspondent abundance of *Escherichia-Shigella* genus. (**C**) Phylogenetic tree of KF843036.1 with HLA-1-1 and top 50 results of Blast from National Center for Biotechnology Information (NCBI). (**D**) Sensitivities of K12 to 1 mM Cd evaluated by 1 × 10^−3^, 10^−4^, and 10^−5^-fold dilution assay: K12 were spotted onto LB media. ^#^, *p* < 0.05 means significantly different compared to the Cd-treated group.

To identify the key species that contributed the most to the *Escherichia-Shigella*, we plotted all the species in this particular genus. From Figure 4A, it is evident that KF843036_g consisted the most among other species. Unfortunately, KF843036_g (registered as KF843036.1 in GenBank from National Center for Biotechnology Information, NCBI) was featured as an “uncultured bacterium clone,” according to NCBI. We then ran a Blast and compared the top 50 hits with KF843036_g. Fortunately, we noticed that the local reserved strain *E. coli* HLA-1-1 (GMDCC NO. 1.2444) exhibits high similarity to KF843036_g (Figure 4B). Most strains of *E. coli* are harmless and contribute to the healthy functioning of the digestive system. A recent study declared that *E. coli* could potentially be used to reduce Cd in wastewater, indicating that *E. coli* may have similar effects *in vivo*. To validate the increasing abundance of *E. coli*, we determined the numbers of *E. coli* in mice feces. The number of *E. coli* increased significantly in the feces of Thr-treated mice in five days (Figure 4C), indicating the large proportion of *E. coli* in the gut.

Next, we examined whether Thr conveys the protective effect on *E. coli* as it did to KF843036_g in GM. In vitro, the protective effect of Thr on *E. coli* K12 under Cd stress was also evaluated. K12 was spotted on Luria-Bertani (LB) plates with/without 1 mM of Cd. The protective effect of Thr against Cd was assessed when K12 was treated with 1 μM of Thr, with Cd resistance observed (Figure 4D). The protective effect of Thr agrees with our previous yeast study. Hence, this study focused on the mechanism of *E. coli* in Cd-exposed mice.

### 2.3. Thr and E. coli Attenuated the Gut Damages Induced by Cd

**Figure 5:**
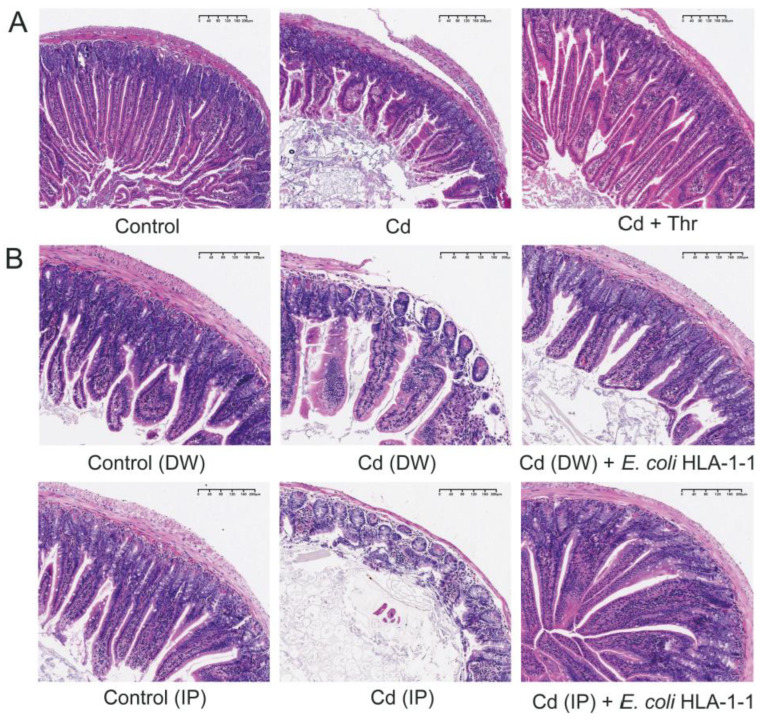
Histopathological changes of the intestine in mice. (**A**) Representative H&E staining photomicrographs of gut sections of mice in control, Cd and Cd + Thr. The magnification of 200 × Bar = 200 μm. (**B**) Representative hematoxylin and eosin staining photomicrographs of gut sections of mice in Control (drinking water, DW), Cd (DW), Cd (DW) + HLA-1-1, Control (intraperitoneal injection, IP), Cd (IP), and Cd (IP) + HLA-1-1 with the magnification of 200 × Bar = 100 μm.

To investigate whether the protective effect of *E. coli* remained consistent with the effect in vitro, we investigated the histopathological changes in the small bowels in Cd-treated mice. In the control group, the intestinal tissue section exhibited normal intestinal morphology with regular-shaped intestinal villi and intact intestinal walls. Incomplete intestinal walls and deciduous villi were found in the Cd-exposed groups. (Figure 5A). However, these gut damages were restored with the oral administration of Thr, with improved integrity of the intestine observed after Thr administration (Figure 5A). The alleviation of gut damage by Thr might also be related to its ability to restore the gut, improve intestinal function, and enhance the diversity of the microbiota, contributing to a direct barrier against toxicants. In Figure 5B, it can be seen that the oral administration of HLA-1-1 restored intestinal integrity and alleviated the level of deciduous villi in mice in both groups that were treated with Cd in drinking water (DW) and intraperitoneally (IP) to varying degrees. These results indicate that the protective effect of Thr and the GM against Cd may be attributed to *E. coli*.

### 2.4. E. coli Facilitated the Excretion of Cd in Mice

**Figure 6:**
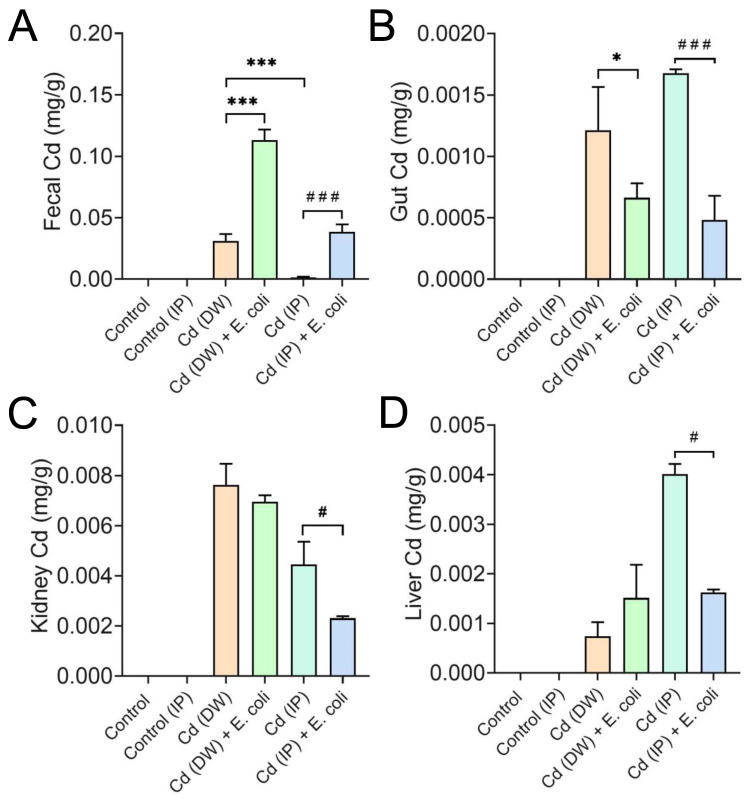
The Contents Cd in feces (**A**), gut (**B**), kidney (**C**), and liver (**D**). * *p* < 0.05, *** *p* < 0.001 indicate results that differ significantly from Cd (DW). ^#^ *p* < 0.05, ^###^ *p* < 0.001 indicate results that differ significantly from Cd (IP). *E. coli* represented HLA-1-1 in this figure.

We next investigated the role of HLA-1-1 playing in the excretion of Cd in Cd-exposed mice. The fecal Cd in the IP group was significantly lower (*p* < 0.05) than that in the DW group, suggesting that the excretion of Cd through feces was more difficult in the IP group (Figure 6A). The oral administration of HLA-1-1 significantly increased (*p* < 0.001) Cd excretion via feces in both the DW and IP groups. In addition, the gut Cd level decreased after oral administration of HLA-1-1 (Figure 6B). This proposes that HLA-1-1 can remove Cd from the host gut. The liver and kidney are the two target organs that accumulate Cd in the organism. It is reported that dysbiosis of the GM leads to the accumulation of Cd in the liver. In this study, 7 days of oral administration of HLA-1-1 significantly reduced the Cd level in the liver and kidney in the IP group (Figure 6C,D). However, the Cd level of the liver increased when treated with Cd (DW) and HLA-1-1 (Figure 6D), indicating that HLA-1-1 was less effective in the DW groups. Taken together, HLA-1-1 exhibited a considerable Cd excretion effect in mice.

## 3. Discussion

Our previous study discovered the unique effect of Thr on mitigating Cd-induced oxidative damage [32]. The present study further explored its potential alleviative effect in Cd-exposed mice, focusing on the first barrier against Cd, the gut [14]. We confirmed that Thr increased the abundance of *E. coli* in the gut to remove Cd.

Among the 20 AAs, Thr was the most effective in removing Cd from mice and decreased the AST and ALT levels in serum, the two biomarkers for evaluating liver oxidative stress [40]. Thr is an important factor concerning the health and productive performance of broiler chicken [41]. Insufficient Thr intake can inhibit the expression of immune genes in prawns [42]. It is reported that the addition of Thr in feed promoted the growth of mice, meanwhile reducing fat accumulation in the liver [43]. Thr was also found to promote the lifespan of *C. elegans* during dietary restriction [44]. In this study, Thr might also promote the growth of the Cd-exposed mice. Given the fact that exposure to Cd is linked to the reduction of Thr content in muscle [45], oral administration of Thr may mitigate such an adverse change. In addition, we previously found the alleviative effect of Thr on the liver under Cd stress [46].

Attempts of *in vivo* Cd removal often focus on the binding features of the agents [47]. Agents with comparatively high Cd binding properties were applied as Cd-removal agents [48]. It tends to imply that Thr carries a great Cd binding property. However, with the relatively poor binding property of Thr detected, we believe that the high Cd excretion in feces fails to be accounted for by simple removal via binding. Instead, as the GM acts as a general barrier in the gut against extraneous threats [49], the restoration of GM induced by Thr sheds light on the potential mechanism of the way of effective Cd removal. From the results of Alpha diversity, UpSet diagram, and NMDS analysis, Thr restored gut microbiota homeostasis. It is noticeable that the Alpha diversity of Asn-treated mice increased dramatically (Figure 1E). While from the later analysis, it is found that the abundance of *Lachnospiraceae* surged among other families, the same family that also went up in the Cd group, similar to the findings of Breton et al. [50]. Asn failed to reverse the Cd-induced changes at the family level as Thr did. Hence, the surge of the abundance is not bound to the restoration of GM. GM can lower intestinal permeability and modulate Cd concentration in the liver [51], indicating that alleviation of Thr to Cd toxicity and Cd removal can be attributed to restoration of the GM.

In this study, we further explored the alteration of the gut microbiota induced by Cd stress and the co-treatment of Cd and Thr. Among the genera restored by Thr, we identified that *Escherichia-Shigella* was the most abundant one, which strongly suggests that it was the key genus in reducing Cd toxicity. According to a present report, diarrhoea-relative symptoms were detected when piglets were challenged with *E. coli* [52]. Nevertheless, not all the species in the *Escherichia-Shigella* were pathogens. Lu et al. [53] reported that *Enterobacter* J1 could absorb Cd. Likewise, *Enterobacter* was also found to absorb Cd and reduce the bioavailability of Cd in rice [54]. *Enterobacter cloacae* can mitigate the detrimental effects of heavy metals like Cd and Ni (nickel) [55]. Hence, we further attempted to determine the exact species of the key microorganism. From the genetical resemblance (16S rDNA), *E. coli* was used for the verification of the Thr protective effect against Cd. As predicted, *E. coli* treated with Thr exhibited a stronger tolerance to Cd stress, which further confirmed the protective effect of Thr on *Escherichia-Shigella*.

Eliminating toxic substances is one of the roles of gut microorganisms, which was demonstrated in vitro previously [56]. The effects of gut microorganisms in vivo have been studied with intestinal bacteria. Cd excretion increased in feces with the oral administration of *Lactobacillus plantarum* strains [57]. In addition, oral administration of *Akkermansia muciniphila* was found to reduce Cd accumulation in the kidneys and livers of mice [57]. *Burkholderia* is able to promote Cd excretion by secreting extracellular polymeric substances [27]. Similar in vivo effects were unveiled in the current study when the GM was treated with Thr, leading to significant improvement in the proportion of *Escherichia-Shigella*, promotion of Cd excretion, and reduction of adverse histopathological changes in guts since fecal excretion is one of the ways for microbiota to detoxify heavy metals [58]. These results provide evidence that *E. coli* is of considerable significance in the reduction of Cd.

In vivo Cd removal effect was verified by orally administrated *E. coli* strains to Cd-exposed mice. *E. coli* exhibited an excellent alleviative effect on Cd toxicity and effectively reduced Cd accumulation. Oral administration of *Lactobacillus plantarum* CCFM8610 was also found to protect Cd-exposed mice [25]. Zhai et al. [25] demonstrated that oral administration of microorganisms reduced Cd-induced damage in the liver and kidneys. Similar results were observed in our study, where oral administration of *E. coli* decreased the Cd contents in the liver and kidneys. However, in terms of reducing Cd concentration in the liver and kidney in the DW groups, *E. coli* was less promising than expected. This may attribute to the fact that a high level of fecal Cd hinders the Cd-adsorption capacity of *E. coli* [59]. The oral administration of HLA-1-1 decreased gut Cd levels but increased fecal Cd levels, implying that HLA-1-1 can facilitate the excretion of gut Cd into the feces.

## 4. Materials and Methods

### 4.1. Reagents and Animals

Cadmium chloride (CdCl_2_, 98%) was purchased from Chengdu Huaxia Chemical Reagent (Chengdu, China). Chrome azurol S (CAS, 98%), 2,2’-Dipyridyl (dipy, 98%), and hexadecylpyridinium bromide (HDPB, 98%) were purchased from Shanghai Acmec Biochemical (Shanghai, China). L-amino acids: alanine (Ala), arginine (Arg), asparagine (Asn), aspartic acid (Asp), cysteine (Cys), glutamic acid (Glu), glutamine (Gln), glycine (Gly), histidine (His), isoleucine (Ile), leucine (Leu), lysine (Lys), methionine (Met), phenylalanine (Phe), proline (Pro), serine (Ser), threonine (Thr), tryptophan (Trp), tyrosine (Tyr) and valine (Val) were purchased from Sangon Biotech Co., Ltd. (Shanghai, China).

Specific pathogen-free (SPF) male Kunming (KM) mice (8 weeks old) were purchased from Tianqin Biotech (Changsha, China) (Production license no. 110324211102567073). The mice were fed with standard commercial rat feed and kept in cages under 12 h light/dark cycles, with free access to feed and water. *Escherichia coli* (*E. coli*) HLA-1-1 (GMDCC NO. 1.2444) was obtained from Guangdong Microbial Culture Collection Center (Guangzhou, China).

The animal experiments were approved by the Laboratory Animal Ethics Committee of Guangdong Ocean University (approval number: GDOU-LAE-2020-009). All animals were treated humanely with a minimum of pain.

### 4.2. Treatment of 20 AAs in Cd-Exposed Mice

A total of 132 SPF mice were randomly divided into 22 groups (Table 1). For the positive control, a dose of 100 μM/L CdCl_2_ in drinking water (DW) was given to mice for 7 days. In the AA-treated groups (Cd (DW) + AA), mice were given drinking water containing 100 μM/L of CdCl_2_ and orally administered 40 μM/g/d of each AA for 7 days. In the control group, mice were orally administered 10 μL/g/d of H_2_O.

### 4.3. Treatment of HLA-1-1 in Cd-Exposed Mice

Thirty-six SPF mice were randomly divided into 6 groups (Table 2). The control group of DW was given H_2_O for 7 days, and the control group of intraperitoneal injection (IP) was intraperitoneally injected with sterilized H_2_O for 7 days. The dose of Cd and HLA-1-1 were set following the study of Qixiao et al. [56]. In Cd (DW) exposure group, mice were given H_2_O containing 100 μM/L of CdCl_2_ for 7 days. In Cd (IP) group, mice were intraperitoneally injected with 9 μg of CdCl_2_ for 7 da 12ys. In Cd (DW) + HLA-1-1 group, mice were given H_2_O containing 100 μmol/L of CdCl_2_ and orally administered with 1 × 10^9^ CFU of HLA-1-1 for 7 days. In Cd (IP) + HLA-1-1 group, mice were intraperitoneally injected with 9 μg of CdCl_2_ and orally administered with 1 × 10^9^ CFU of HLA-1-1 for 7 days.

### 4.4. Determination of Cd Contents in Feces and Tissues

The Cd contents in feces and tissues were determined as previously described [56]. The fecal samples from each group were collected every day. Heart, liver, spleen, lung, and kidney were collected when the mice were sacrificed. The fecal and tissue samples were ground evenly and digested with HNO_3_/H_2_O_2_ (5:1, *v*/*v*) mixture using a microwave digestion system (Multiwave PRO 41HV56, Anton-paar, Shanghai, China). Cd content was measured by SK-Ruixi AFS atomic fluorescence spectrometer (Shanghai, China).

### 4.5. Determination of Conductivity Difference

Metal chelation with AAs results in a change in conductivity [32]. 1 mM of 20 AAs was added into 0.5 M CdCl_2_ for the determination of conductivity. The difference in conductivity was calculated as Δσ = A − D + (B − D) − (C − D). A is the conductivity of the CdCl_2_ solution; B is the conductivity of the AA solution; C is the conductivity of the CdCl_2_ + AA solution; D is the conductivity of Hydroxyethylpiperazine Ethane Sulfonic Acid (HEPES, 4-(2-hydroxyethyl)-1-piperazineethanesulfonic acid).

### 4.6. 16S rDNA Gene Sequencing of GM

16S rDNA was sequenced as previously described [60]. The total bacterial genomic DNA was extracted from fresh mice feces in each group by PowerMax (stool/soil) DNA isolation kit (MoBioLaboratories, Carlsbad, CA, USA). NanoDrop ND-1000 spectrophotometer (Thermo Fisher Scientific, Waltham, MA, USA) was used for quantitative measurement, while agarose gel electrophoresis was used for quality measurement. Forward primer 515F (5′-GTGCCAGCMGCCGCGGTAA-3′) and reverse primer 806R (5′-GGACTACHVGGGTWTCTAAT-3′) were targeted in the V4 region of bacterial 16S rDNA [61].

Sequencing data was processed by the Quantitative Insights Into Microbial Ecology (QIIME, v1.9.0) pipeline [62]. Raw reads with exact matches to the barcodes were assigned to respective samples and identified as valid sequences. Filtered the low-quality reads as previously described [63]. Vsearch V2.4.4 was employed to assemble the paired-end reads and pick to an operational taxonomic unit (OTU). QIIME and R packages (v3.2.0) were applied for further data analyses. Heatmap was generated by TB tools [64]. Bioinformatic analysis of the upset diagram, circos graph, and correlation heatmap was performed using the OmicStudio tools at https://www.omicstudio.cn/tool (accessed on 23 December 2022). Alignment of the 16S rDNA and plotting of the phylogenetic tree were calculated and generated in Clustal Omega (https://www.ebi.ac.uk/Tools/msa/clustalo/, accessed on 23 December 2022).

### 4.7. Histopathological Examination

Gut sections were collected when the mice were sacrificed. Gut sections were sampled from the representative small bowels (chosen randomly). A part of the small bowel was fixed with 4% paraformaldehyde universality fixative, overnight at 4 °C, embedded with paraffin, and sectioned into 5 μm sections as previously described [65]. The sections were stained with hematoxylin and eosin (H&E) and then subjected to microscope analysis for damage to the intestinal villus and the integrity of the intestinal wall.

### 4.8. Determination of Cd-Chelating Ability

A modified CAS liquid assay for the determination of Cd-chelating ability was improved from a Fe-CAS assay [66], which is conducted as follows: 4 mL 50 mM CAS, 2 mL 10 mM dipy, 1.5 mL 1 × 10^−3^ M HDPB, and 100 mM NaOH was titrated till the system turned green. After being placed stably for 5 min, a 5 mL buffer solution of sodium borate sodium hydroxide (pH 11.0) was added. Then diluted with H_2_O to a constant volume of 25 mL. The change of optical density value (OD) was measured at 602 nm within 40 min using a Varioskan Flash Multiplate Reader (Thermo Fisher, MA, USA). For the CAS plate assay, 2 g/100 mL agar was added to the solution system mentioned above. The chelation rate was calculated as (H_2_O-treated OD value–treatment OD)/(H_2_O-treated OD value). 

Twenty AAs (1 mM) were respectively added to the CAS reaction system to determine the OD value. HLA-1-1 was cultured for 7 days with/without Thr to obtain the supernatant after centrifuging at 10,000 r/min for 10 min, then stored at −50 °C targeting concentrate supernatant via vacuum freeze-drying. Strains were harvested from fragments of HLA-1-1 obtained from the culture media after centrifuging at 10 000 r/min for 10 min, then put in the bead beater for milling a couple of times after adding magnetic beads.

### 4.9. Cd-Sensitivity Assay for E. coli

Thr and K12 were cultured in LB media for 24 h at 37 °C to evaluate the protective effect of Thr. Spot assay was performed as previously described to evaluate gene sensitivity [32]. K12 was serially diluted as 1 × 10^−3^, 10^−4^, and 10^−5^-folds after the cell density reached OD600 of 1. 3 μL of dilution of K12 was spotted onto LB plates containing 0 mM CdCl_2_, 1 mM CdCl_2_, and 1 mM CdCl_2_ + 1 μM of Thr to incubated for 24 h.

## 5. Conclusions

In summary, among the 20 AAs, Thr was the most effective in increasing the fecal levels of Cd and was able to restore the *Escherichia-Shigella* genus. In this study, the potential availability and mechanism underlying the ability of Thr to reduce Cd accumulation by effectively facilitating Cd excretion in feces were well demonstrated. Thr restored the *Escherichia-Shigella* genus under Cd stress and *E. coli* removed Cd in vivo, alleviating Cd-induced damage to multiple organs. This study shows that Thr can be used as a supplementary ingredient to protect against Cd toxicity. Further studies are now underway to better understand the molecular biological mechanism underlying the ability of Thr to actively reduce gut Cd.

## Figures and Tables

**Table 1:** Primary experimental protocol of Cd-exposed mice treated with amino acids.

Groups	*n*	Concentration of Cd in Drinking Water (μM) ^a^	Oral Administration (mmol/g/d bw) ^b^
Control	6	-	-
Cd (DW) ^c^	6	100	-
Cd (DW) + AA	6 × 20	100	40 (AA)

^a^ The daily volume of drinking water and Cd water were 150 mL; ^b^ AA were given ever since the first day via intragastric administration for 7 d; ^c^ DW means that mice were exposed to Cd via drinking water containing 0 or 100 μM CdCl_2_.

**Table 2:** Experimental protocol of Cd-exposed mice treated with *E. coli* (HLA-1-1).

Groups	*n*	Concentration of Cd in Drinking Water (μM)	Intraperitoneal Injection of Cd (μg/d)	Oral Administration of *E. coli* (CFU/d)
Control (DW)	6	-	-	-
Control (IP)	6	-	-	-
Cd (DW)	6	100	-	-
Cd (IP)	6	-	9	-
Cd (DW) + *E. coli*	6	100	-	1 × 10^9^
Cd (IP) + *E. coli*	6	-	9	1 × 10^9^

## Data Availability

The data that support the findings of this study are available from the corresponding authors upon reasonable request.

## Sample Availability

Samples of the compounds are not available from the authors.

## References

[1] Shi J., Du P., Luo H., Wu H., Zhang Y., Chen J., Wu M., Xu G., Gao H. (2022). Soil contamination with cadmium and potential risk around various mines in China during 2000–2020. J. Environ. Manag..

[2] Jiang Y., Fei J., Cao P., Zhang C., Tang M., Cheng J., Zhao H., Fu L. (2021). Serum cadmium positively correlates with inflammatory cytokines in patients with chronic obstructive pulmonary disease. Environ. Toxicol..

[3] Fernández-Torres J., Zamudio-Cuevas Y., Martínez-Nava G.A., Aztatzi-Aguilar O.G., Sierra-Vargas M.P., Lozada-Pérez C.A., Suárez-Ahedo C., Landa-Solís C., Olivos-Meza A., Del Razo L.M. (2021). Impact of Cadmium Mediated by Tobacco Use in Musculoskeletal Diseases. Biol. Trace Element Res..

[4] Ma S., Zhang J., Xu C., Da M., Xu Y., Chen Y., Mo X. (2021). Increased serum levels of cadmium are associated with an elevated risk of cardiovascular disease in adults. Environ. Sci. Pollut. Res..

[5] Satarug S., Đorđević A.B., Yimthiang S., Vesey D.A., Gobe G.C. (2022). The NOAEL Equivalent of Environmental Cadmium Exposure Associated with GFR Reduction and Chronic Kidney Disease. Toxics.

[6] Pan J., Plant J.A., Voulvoulis N., Oates C.J., Ihlenfeld C. (2010). Cadmium levels in Europe: Implications for human health. Environ. Geochem. Health.

[7] Ngugi M.M., Gitari H.I., Muii C., Gweyi-Onyango J.P. (2022). Cadmium mobility, uptake, and accumulation in spinach, kale, and amaranths vegetables as influenced by silicon fertilization. Bioremediation J..

[8] Elhelaly A.E., Elbadry S., Eltanani G.S.A., Saad M.F., Darwish W.S., Tahoun A.B.M.B., Ellatif S.S.A. (2022). Residual contents of the toxic metals (lead and cadmium), and the trace elements (copper and zinc) in the bovine meat and dairy products: Residues, dietary intakes, and their health risk assessment. Toxin Rev..

[9] Cui S., Wang Z., Li X., Wang H., Wang H., Chen W. (2022). A comprehensive assessment of heavy metal(loid) contamination in leafy vegetables grown in two mining areas in Yunnan, China—A focus on bioaccumulation of cadmium in Malabar spinach. Environ. Sci. Pollut. Res..

[10] Spungen J.H. (2019). Children’s exposures to lead and cadmium: FDA total diet study 2014–16. Food Addit. Contam. Part A.

[11] Johri N., Jacquillet G., Unwin R. (2010). Heavy metal poisoning: The effects of cadmium on the kidney. Biometals.

[12] Park J.D., Cherrington N.J., Klaassen C.D. (2002). Intestinal Absorption of Cadmium Is Associated with Divalent Metal Transporter 1 in Rats. Toxicol. Sci..

[13] He X., Qi Z., Hou H., Qian L., Gao J., Zhang X.-X. (2020). Structural and functional alterations of gut microbiome in mice induced by chronic cadmium exposure. Chemosphere.

[14] He X., Qi Z., Hou H., Gao J., Zhang X.-X. (2020). Effects of chronic cadmium exposure at food limitation-relevant levels on energy metabolism in mice. J. Hazard. Mater..

[15] Zhu G., Cheng D., Wang X., Guo Q., Zhang Q., Zhang J., Tu Q., Li W. (2022). Free amino acids, carbon and nitrogen isotopic compositions responses to cadmium stress in two castor (*Ricinus communis* L.) species. Plant Physiol. Biochem..

[16] Su X., Gao Y., Yang R. (2022). Gut microbiota-derived tryptophan metabolites maintain gut and systemic homeostasis. Cells.

[17] Wozniak H., Beckmann T.S., Fröhlich L., Soccorsi T., Le Terrier C., de Watteville A., Schrenzel J., Heidegger C.-P. (2022). The central and biodynamic role of gut microbiota in critically ill patients. Crit. Care.

[18] Zhang X., Gérard P. (2022). Diet-gut microbiota interactions on cardiovascular disease. Comput. Struct. Biotechnol. J..

[19] Schlechte J., Skalosky I., Geuking M.B., McDonald B. (2022). Long-distance relationships—Regulation of systemic host defense against infections by the gut microbiota. Mucosal Immunol..

[20] Kamioka M., Goto Y., Nakamura K., Yokoi Y., Sugimoto R., Ohira S., Kurashima Y., Umemoto S., Sato S., Kunisawa J. (2022). Intestinal commensal microbiota and cytokines regulate Fut2 ^+^ Paneth cells for gut defense. Proc. Natl. Acad. Sci. USA.

[21] Antunes L.C.M., McDonald J.A.K., Schroeter K., Carlucci C., Ferreira R.B.R., Wang M., Yurist-Doutsch S., Hira G., Jacobson K., Davies J. (2014). Antivirulence Activity of the Human Gut Metabolome. MBio.

[22] Duan H., Yu L., Tian F., Zhai Q., Fan L., Chen W. (2020). Gut microbiota: A target for heavy metal toxicity and a probiotic protective strategy. Sci. Total. Environ..

[23] Feng P., Yang J., Zhao S., Ling Z., Han R., Wu Y., Salama E.-S., Kakade A., Khan A., Jin W. (2022). Human supplementation with Pediococcus acidilactici GR-1 decreases heavy metals levels through modifying the gut microbiota and metabolome. NPJ Biofilms Microbiomes.

[24] Breton J.Ô., Daniel C., Dewulf J., Pothion S., Froux N., Sauty M., Thomas P., Pot B., Foligne B. (2013). Gut microbiota limits heavy metals burden caused by chronic oral exposure. Toxicol. Lett..

[25] Zhai Q., Tian F., Zhao J., Zhang H., Narbad A., Chen W. (2016). Oral Administration of Probiotics Inhibits Absorption of the Heavy Metal Cadmium by Protecting the Intestinal Barrier. Appl. Environ. Microbiol..

[26] Fazeli M., Hassanzadeh P., Alaei S. (2011). Cadmium chloride exhibits a profound toxic effect on bacterial microflora of the mice gastrointestinal tract. Hum. Exp. Toxicol..

[27] Fang Z., Chen Y., Li Y., Sun L., Deng Q., Wang J., Gooneratne R. (2022). Oleic Acid Facilitates Cd Excretion by Increasing the Abundance of *Burkholderia* in Cd-Exposed Mice. Int. J. Mol. Sci..

[28] Kumar N., Kumar V., Panwar R., Ram C. (2017). Efficacy of indigenous probiotic Lactobacillus strains to reduce cadmium bioaccessibility—An in vitro digestion model. Environ. Sci. Pollut. Res..

[29] Zhai Q., Narbad A., Chen W. (2015). Dietary strategies for the treatment of cadmium and lead toxicity. Nutrients.

[30] Sun S., Zhou X., Li Y., Li Y., Xia H., Li Z., Zhuang P. (2020). Use of dietary components to reduce the bioaccessibility and bioavailability of cadmium in rice. J. Agric. Food Chem..

[31] Rafieian-Naeini H.R., Zhandi M., Sadeghi M., Yousefi A.R., Marzban H., Benson A.P. (2022). The effect of dietary coenzyme Q10 supplementation on egg quality and liver histopathology of layer quails under cadmium challenge. J. Anim. Physiol. Anim. Nutr..

[32] Linru H., Zhijia F., Jian G., Jingwen W., Yongbin L., Lijun S., Yaling W., Jianmeng L., Ravi G. (2021). Protective role of l -threonine against cadmium toxicity in *Saccharomyces cerevisiae*. J. Basic Microbiol..

[33] Rafiq S., Huma N., Pasha I., Sameen A., Mukhtar O., Khan M.I. (2015). Chemical Composition, Nitrogen Fractions and Amino Acids Profile of Milk from Different Animal Species. Asian-Australas. J. Anim. Sci..

[34] Ni F., Yu W.-M., Li Z., Graham D.K., Jin L., Kang S., Rossi M.R., Li S., Broxmeyer H.E., Qu C.-K. (2019). Critical role of ASCT2-mediated amino acid metabolism in promoting leukaemia development and progression. Nat. Metab..

[35] Zemanová V., Pavlík M., Pavlíková D., Tlustoš P. (2014). The significance of methionine, histidine and tryptophan in plant responses and adaptation to cadmium stress. Plant Soil Environ..

[36] Paniagua-Castro N., Escalona-Cardoso G., Cevallos G.C. (2007). Glycine reduces cadmium-induced teratogenic damage in mice. Reprod. Toxicol..

[37] Hwang D.F., Wang L.C. (2001). Effect of taurine on toxicity of cadmium in rats. Toxicology.

[38] Bifari F., Ruocco C., Decimo I., Fumagalli G., Valerio A., Nisoli E. (2017). Amino acid supplements and metabolic health: A potential interplay between intestinal microbiota and systems control. Genes Nutr..

[39] Kojima S., Kaminaka K., Kiyozumi M., Honda T. (1986). Comparative effects of three chelating agents on distribution and excretion of cadmium in rats. Toxicol. Appl. Pharmacol..

[40] Firouzian F., Pourshoja P., Nili-Ahmadabadi A., Ranjbar A. (2019). Hepatoprotective effect of N-acetylcystein loaded niosomes on liver function in paraquat-induced acute poisoning. Pestic. Biochem. Physiol..

[41] Nabi F., Arain M.A., Bhutto Z.A., Shah Q.A., Bangulzai N., Ujjan N.A., Fazlani S.A. (2022). Effect of early feeding of L-arginine and L-threonine on hatchability and post-hatch performance of broiler chicken. Trop. Anim. Health Prod..

[42] Worlanyo H.G., Jiang S., Yu Y., Liu B., Zhou Q., Sun C., Miao L., Lin Y., Zheng X., Saidyleigh M. (2022). Effects of dietary threonine on growth and immune response of oriental river prawn (*Macrobrachium nipponense*). Fish Shellfish Immunol..

[43] Harper A.E., Monson W.J., Benton D.A., Elvehjem C.A. (1953). The Influence of Protein and Certain Amino Acids, Particularly Threonine, on the Deposition of Fat in the Liver of the Rat. J. Nutr..

[44] Kim J., Jo Y., Cho D., Ryu D. (2022). L-threonine promotes healthspan by expediting ferritin-dependent ferroptosis inhibition in *C. elegans*. Nat. Commun..

[45] Daabees A.Y. (1987). Portubations of amino acid levels in plasma and liver of rat by cadmium chloride. Bull. Fac. Sci. Univ. Alex..

[46] Fang Z., Li Y., Wang J., Wang X., Huang L., Sun L., Deng Q. (2022). Alleviative effect of threonine on cadmium-induced liver injury in mice. Biol. Trace Element Res..

[47] Daisley B.A., Monachese M., Trinder M., Bisanz J.E., Chmiel J.A., Burton J.P., Reid G. (2019). Immobilization of cadmium and lead by Lactobacillus rhamnosus GR-1 mitigates apical-to-basolateral heavy metal translocation in a Caco-2 model of the intestinal epithelium. Gut Microbes.

[48] Zhai Q., Yin R., Yu L., Wang G., Tian F., Yu R., Zhao J., Liu X., Chen Y.Q., Zhang H. (2015). Screening of lactic acid bacteria with potential protective effects against cadmium toxicity. Food Control..

[49] Jakobsson H.E., Rodríguez-Piñeiro A.M., Schütte A., Ermund A., Boysen P., Bemark M., Sommer F., Bäckhed F., Hansson G.C., Johansson M.E.V. (2015). The composition of the gut microbiota shapes the colon mucus barrier. EMBO Rep..

[50] Breton J., Massart S., Vandamme P., De Brandt E., Pot B., Foligné B. (2013). Ecotoxicology inside the gut: Impact of heavy metals on the mouse microbiome. BMC Pharmacol. Toxicol..

[51] Liu Y., Li Y., Xia Y., Liu K., Ren L., Ji Y. (2020). The dysbiosis of gut microbiota caused by low-dose cadmium aggravate the injury of mice liver through increasing intestinal permeability. Microorganisms.

[52] Jiménez M.J., Berrios R., Stelzhammer S., Hohmann M., Verri W., Bracarense A.P.F.R.L. (2020). Ingestion of organic acids and cinnamaldehyde improves tissue homeostasis of piglets exposed to enterotoxic *Escherichia coli* (ETEC). J. Anim. Sci..

[53] Lu W.-B., Shi J.-J., Wang C.-H., Chang J.-S. (2006). Biosorption of lead, copper and cadmium by an indigenous isolate Enterobacter sp. J1 possessing high heavy-metal resistance. J. Hazard. Mater..

[54] Mitra S., Pramanik K., Sarkar A., Ghosh P.K., Soren T., Maiti T.K. (2018). Bioaccumulation of cadmium by *Enterobacter* sp. and enhancement of rice seedling growth under cadmium stress. Ecotoxicol. Environ. Saf..

[55] Badawy I.H., Hmed A.A., Sofy M.R., Al-Mokadem A.Z. (2022). Alleviation of cadmium and nickel toxicity and phyto-stimulation of tomato plant l. by endophytic *micrococcus luteus* and *enterobacter cloacae*. Plants.

[56] Zhai Q., Wang G., Zhao J., Liu X., Narbad A., Chen Y.Q., Zhang H., Tian F., Chen W. (2014). Protective Effects of Lactobacillus plantarum CCFM8610 against Chronic Cadmium Toxicity in Mice Indicate Routes of Protection besides Intestinal Sequestration. Appl. Environ. Microbiol..

[57] Zhai Q., Liu Y., Wang C., Zhao J., Zhang H., Tian F., Lee Y.-k., Chen W. (2019). Increased cadmium excretion due to oral administration of lactobacillus plantarum strains by regulating enterohepatic circulation in mice. J. Agric. Food Chem..

[58] Liu T., Liang X., Lei C., Huang Q., Song W., Fang R., Li C., Li X., Mo H., Sun N. (2020). High-Fat Diet Affects Heavy Metal Accumulation and Toxicity to Mice Liver and Kidney Probably via Gut Microbiota. Front. Microbiol..

[59] Laddaga R.A., Silver S. (1985). Cadmium uptake in *Escherichia coli* K-12. J. Bacteriol..

[60] Wang R., Deng Y., Deng Q., Sun D., Fang Z., Sun L., Wang Y., Gooneratne R. (2020). *Vibrio parahaemolyticus* infection in mice reduces protective gut microbiota, Augmenting Disease Pathways. Front. Microbiol..

[61] Shang Q., Song G., Zhang M., Shi J., Xu C., Hao J., Li G., Yu G. (2017). Dietary fucoidan improves metabolic syndrome in association with increased Akkermansia population in the gut microbiota of high-fat diet-fed mice. J. Funct. Foods.

[62] Caporaso J.G., Kuczynski J., Stombaugh J., Bittinger K., Bushman F.D., Costello E.K., Fierer N., Peña A.G., Goodrich J.K., Gordon J.I. (2010). QIIME allows analysis of high-throughput community sequencing data. Nat. Methods.

[63] Chen H., Jiang W. (2014). Application of high-throughput sequencing in understanding human oral microbiome related with health and disease. Front. Microbiol..

[64] Chen C.J., Chen H., Zhang Y., Thomas H.R., Frank M.H., He Y.H., Xia R. (2020). TBtools: An Integrative Toolkit Developed for Interactive Analyses of Big Biological Data. Mol. Plant.

[65] Chen S., Zheng Y., Zhou Y., Guo W., Tang Q., Rong G., Hu W., Tang J., Luo H. (2019). Gut Dysbiosis with Minimal Enteritis Induced by High Temperature and Humidity. Sci. Rep..

[66] Xiao X., Yeoh B.S., Saha P., Tian Y., Singh V., Patterson A.D., Vijay-Kumar M. (2016). Modulation of urinary siderophores by the diet, gut microbiota and inflammation in mice. J. Nutr. Biochem..

